# Hypoxic Culture Conditions as a Solution for Mesenchymal Stem Cell Based Regenerative Therapy

**DOI:** 10.1155/2013/632972

**Published:** 2013-08-27

**Authors:** Nazmul Haque, Mohammad Tariqur Rahman, Noor Hayaty Abu Kasim, Aied Mohammed Alabsi

**Affiliations:** ^1^Regenerative Dentistry Research Group, Faculty of Dentistry, University of Malaya, 50603 Kuala Lumpur, Malaysia; ^2^Department of Conservative Dentistry, Faculty of Dentistry, University of Malaya, 50603 Kuala Lumpur, Malaysia; ^3^Department of Biotechnology, Faculty of Science, International Islamic University Malaysia, 25200 Kuantan, Malaysia; ^4^Dental Research and Training Unit, Faculty of Dentistry, University of Malaya, 50603 Kuala Lumpur, Malaysia; ^5^Oral Cancer Research & Coordinating Centre, Faculty of Dentistry, University of Malaya, 50603 Kuala Lumpur, Malaysia

## Abstract

Cell-based regenerative therapies, based on *in vitro* propagation of stem cells, offer tremendous hope to many individuals suffering from degenerative diseases that were previously deemed untreatable. Due to the self-renewal capacity, multilineage potential, and immunosuppressive property, mesenchymal stem cells (MSCs) are considered as an attractive source of stem cells for regenerative therapies. However, poor growth kinetics, early senescence, and genetic instability during *in vitro* expansion and poor engraftment after transplantation are considered to be among the major disadvantages of MSC-based regenerative therapies. A number of complex inter- and intracellular interactive signaling systems control growth, multiplication, and differentiation of MSCs in their niche. Common laboratory conditions for stem cell culture involve ambient O_2_ concentration (20%) in contrast to their niche where they usually reside in 2–9% O_2_. Notably, O_2_ plays an important role in maintaining stem cell fate in terms of proliferation and differentiation, by regulating hypoxia-inducible factor-1 (HIF-1) mediated expression of different genes. This paper aims to describe and compare the role of normoxia (20% O_2_) and hypoxia (2–9% O_2_) on the biology of MSCs. Finally it is concluded that a hypoxic environment can greatly improve growth kinetics, genetic stability, and expression of chemokine receptors during *in vitro* expansion and eventually can increase efficiency of MSC-based regenerative therapies.

## 1. Introduction 

The promising role of stem cell therapy is becoming more conceivable in addressing the unmet needs of treating degenerative diseases through conventional medicine. Diseases such as diabetes, myocardial infarction, spinal cord injury, stroke, and Parkinson's and Alzheimer's diseases have become more prevalent with increasing life expectancy. It has been estimated that in the United States alone, approximately 128 million individuals would benefit from regenerative stem cell therapy during their lifetime [1].

Self-renewal and multipotency are the key hallmarks of stem cells, permitting them to act as the fundamental units maintaining growth, homeostasis and repair of many tissues. These two key features establish stem cells as the most promising tool for regenerative medicine [2, 3]. Among the different types of stem cells, mesenchymal stem cells (MSCs) or multipotent mesenchymal stromal cells [4] are considered as a potential tool to treat degenerative diseases. This is due to their multipotent differentiative capacity [5–7] with trophic activity [8, 9], potent immunosuppressive effects [10–12], and ability to induce vascularisation [13]. Moreover, MSCs can be efficiently isolated from tissues such as bone marrow, adipose tissue, umbilical cord, and dental pulp [14–17]. These properties have fascinated and encouraged researchers to push the frontiers of regenerative medicine, utilizing MSCs to treat a large variety of pathologies, including traumatic lesions, stroke, autoimmune diseases, musculoskeletal and cardiac disorders [18–21]. 

Despite the various sources, concentration of MSCs within tissues is very low [22, 23], and it is not possible to isolate 50–200 million MSCs (typically used in clinical trials) from a donor for each therapy [24–29]. Thus, *in vitro* expansion of MSCs has become an inevitable option [23]. In several clinical trials, MSCs expanded *in vitro* are being transplanted to find out their efficacy in treating degenerative diseases, reducing acute rejection of transplanted organs, and in preventing and treating graft-versus-host disease [25, 29–32]. Sometimes the expanded cells are induced to differentiate into a particular cell type and then the predifferentiated cells are transplanted for the regeneration of particular tissues or organs [33]. After transplantation, tissue-specific migration and engraftment ensure the success of cell-based regenerative therapy. 

**Figure 1:**
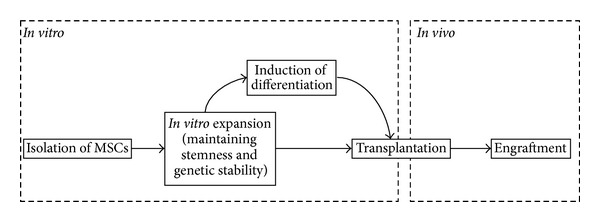
Steps involved in MSCs-based therapy.

From isolation to engraftment, the MSCs usually pass through two different environmental conditions. One is the *in vitro* culture condition (from isolation to transplantation) and the other is the *in vivo* or physiological condition (before isolation and after transplantation) (Figure 1). At present, most of the expansion procedures of MSCs are performed under ambient O_2_ concentration, where cells are exposed to 20% O_2_, which is approximately 4–10 times more than the concentration of O_2_ in their natural niches [35, 36]. The higher O_2_ concentration might cause environmental stress to the *in vitro* cultured MSCs. Moreover, in recent years, several studies have presented clear evidence regarding the negative influence of ambient O_2_ concentration on MSCs, including early senescence, longer population doubling time, DNA damage [37, 38], and poor engraftment following transplantation [33, 39]. All these have shown the influential effect of O_2_ concentration on MSCs biology and raised serious concern over its therapeutic efficiency and biosafety.

Numerous *in vitro* studies have been conducted in the last two decades to analyze the complex processes involved in stem cell maintenance. However, the role of physiologically normoxic (hypoxic) conditions (usually 2–9% O_2_ concentration) on stem cell biology received very little attention [40]. Thus, this paper discusses the differences between *in vitro* MSC culture in ambient and hypoxic conditions. Finally this paper also highlights how MSCs cultured* in vitro *in hypoxic conditions can offer a solution for MSCs-based therapy. 

## 2. Stem Cell Niche

In both *in vitro* and *in vivo* conditions, the fate and function of stem cells depend upon their intrinsic genetic program and the local microenvironment, often referred to as the “stem cell niche” [41]. The stem cell niche concept was proposed by Schofield in 1978 [42], and several researchers have tried to elucidate the confusion and controversy over it [43–45]. “Stem cell niche” can be defined as the anatomical compartment composed of cellular and acellular components that orchestrate both systemic and local signals to control the rate of stem cell proliferation, to determine the fate of stem cell daughters, and to protect stem cells from exhaustion or death [46–48]. The cellular and acellular components of the stem cell niche can be divided into four main groups of key factors, namely, the regulatory molecules (O_2_, nutrients, and cytokines), other cells (3D context, cell-cell contacts, autocrine, and paracrine signals), extracellular matrix (immobilized and released factors, structure, topology, and stiffness), and physical factors (flow shear, compression, stretch, and electrical signals) [41]. 

### 2.1. The Hypoxic Embryonic Stem Cell Niche

In mammals, from fertilization to parturition, cells within the embryo face continuously change in O_2_ concentration [49]. During the time of blastocyst implantation, O_2_ levels within the lumen of the uterus remain as low as 1-2% [50]. In human tissues, O_2_ has a diffusion distance of approximately 150 *μ*m [51, 52], which regulates the O_2_ supply during development and implantation of the blastocyst [53]. However, after development of the circulatory system until 8–10 weeks of gestation, the placental O_2_ levels remain lower (approximately 2-3%) than those in the surrounding endometrium and reach physiological O_2_ concentration at the 12-13th week of gestation [54, 55]. Therefore, embryos go through hypoxic O_2_ concentrations while passing through different developmental periods. Among all the embryonic stages, blastocyst which resides in a hypoxic environment has been recognized as the main source of pluripotent embryonic stem cells (ESCs). 

Recently, a new type of pluripotent stem cell has been generated by reprogramming human adult somatic cells. Pluripotency of this cell type is comparable to human ESCs and commonly referred as “induced pluripotent stem cells” (iPSCs) [56]. Hypoxic culture environments have shown to enhance the generation of these iPSCs too [57, 58]. 

### 2.2. The Hypoxic Environment of the Mesenchymal Stem Cell Niche

Like ESCs, MSCs also reside in low O_2_ concentrations. In mammals including humans, MSCs are located in perivascular niches close to the vascular structure in almost all tissues [17, 59, 60]. Despite residing near the blood vessels, in different tissues where they are found, the O_2_ concentrations are low [61, 62]. In adult human tissues, O_2_ concentration varies widely (Table 1) depending on the vascularisation and the type of microenvironment within the respective organ, and they are considerably lower than the inhaled ambient O_2_ concentration (21%). The partial pressure or O_2_ concentration of inspired air gradually decreases after it enters the lungs and then in the blood flowing from the alveolar capillaries that carry O_2_, towards the organs and tissues for their oxygenation. By the time O_2_ reaches the organs and tissues, O_2_ concentration drops to 2%–9%, with a mean of 3% [40, 63].

As the concentrations of O_2_ in blastocysts and the MSCs niches are very low [73, 76, 77], this could be an important clue for maintaining the self-renewal property and plasticity of MSCs.

## 3. Comparison between Culture in Hypoxic and Ambient Environments

Since 1963, when the isolation and self-renewing properties of mouse bone marrow cells were first reported [78, 79], until now most of the research efforts have been focused on the identification of molecular markers [4, 80, 81]. This has allowed the isolation of different types of tissue-specific stem or progenitor cells [82–85] and has also assisted to define the differentiation of stem or progenitor cells into a particular cell type [86, 87]. Moreover, the development of specific methods for functional stem cell isolation and identification is highly important, in order to study the molecular mechanisms behind the multipotentiality and self-renewable capacity of stem cells and also for the establishment of stem cell-based regenerative therapeutics. This trend has overshadowed the importance of O_2_ concentration, a key environmental factor that might play a vital role on stem cell fate and function [40]. Unfortunately till now in most laboratories, stem cells are typically cultured under the ambient O_2_ concentration without paying attention to the metabolic milieu of the niche in which they grow or normally reside [88]. However, in recent years, scientists have started to manipulate the O_2_ concentration in cell cultures by maintaining a niche-like hypoxic environment. Though the effect of hypoxic culture conditions on the proliferation and differentiation potential of MSCs has been reviewed by few researchers [77, 89], the effect of hypoxia on the genetic stability, early senescence, and site-specific migration of MSCs has not been reviewed in depth. Thus, on the basis of recent research outcomes, the effect of different O_2_ concentration on MSCs biology is further discussed.

### 3.1. Proliferation of MSCs

Capability for self-renewal is a key feature of stem cells. An increased proliferation rate is necessary for more efficient use of stem cells in regenerative therapies. Fehrer et al. (2007) demonstrated that bone marrow-derived MSCs (BM-MSCs) cultured in 3% O_2_ concentration showed significantly increased *in vitro* proliferative lifespan, with approximately 10 additional population doublings (PDs) (28.5 ± 3.8 PD in 20% O_2_ and 37.5 ± 3.4 PD in 3% O_2_) before reaching senescence compared to cells cultured in the ambient O_2_ environment [38]. In addition, early passaged MSCs cultured in hypoxic conditions also exhibit increased proliferative lifespan along with significant difference in population doubling [37]. Furthermore, it is possible to harvest more than 1 × 10^9^ MSCs from the first five passages cultured in 3% O_2_, whereas in ambient condition only 2 × 10^7^ cells can be obtained [37]. Higher *in vitro* expansion rate in hypoxic conditions has also been reported by several other researchers [90–93]. Such *in vitro* culture environment also allows to maintain a higher proportion of rapidly self-renewing MSCs for a longer period of time [94]. However, proliferation of MSCs was reduced significantly in 1% or less O_2_ concentration [95]. 

### 3.2. Plasticity of MSCs

Besides higher growth kinetics, maintaining plasticity is also an important factor for prospective use of MSCs in regenerative medicine. Trilineage (osteogenic, chondrogenic, and adipogenic) mesenchymal differentiation is a unique biological property of MSCs [4]. Several researchers reported the effect of different culture O_2_ concentrations on the trilineage differentiation of MSCs. In an elegantly designed experiment, Raheja et al. (2010) seeded and induced MSCs for differentiation under an atmosphere of 5% carbon dioxide (CO_2_) along with 1 of 4 O_2_ concentrations (1%, 2%, 5%, and 21%). According to their results, MSCs differentiated into osteoblast most rapidly in 21% O_2_, and O_2_ below 5% showed reduced differentiation potential. However, no statistically significant difference in osteogenic marker was reported when O_2_ was between 5% and 21% [96]. In addition, Basciano et al. (2011) have reported improved osteoblastic and adipogenic differentiation potential of early passaged (P2) MSCs in 5% O_2_ concentration [90]. Several other recent reports support that the multilineage differentiation potential of MSCs can be maintained under hypoxic (1–5% O_2_ concentration) environment [91, 92, 95, 97]. Increased adipogenic and osteogenic differentiation potentials of adipose tissue-derived MSCs precultured in hypoxic environment have also been reported [98]. In contrast, few researchers showed reduction in the differentiation potential of MSCs when maintained and induced for differentiation in 1% O_2_ concentration [99, 100].

### 3.3. Genetic Stability of MSCs

Genetic instability of MSCs is another major problem that is directly related to the biosafety of stem cell therapy. For instance, aneuploidy, DNA breakdown, and telomere shortening can be observed in cultured MSCs [37, 101, 102]. However, Tarte et al. (2010) reported that aneuploidy in cultured MSCs is donor dependent rather than its dependence on the culture environment [102]. In contrast, Estrada et al. (2012) have shown a negative effect of ambient O_2_ concentration on cultured MSCs responsible in bringing about DNA damage and aneuploidy. However, this effect was minimized by expanding MSCs in a physiological O_2_ concentration [37]. There is scientific evidence that aneuploidy is a major cause of tumorigenesis [103, 104] which raised concerns regarding the biosafety of MSCs cultured in ambient O_2_ condition.

### 3.4. Engraftment of MSCs

Engraftment is an important part of MSC therapy. Modest engraftment capacity following transplantation of MSCs cultured in ambient condition has been reported in some clinical trial reports [33, 39]. Unpretentious therapeutic outcomes of clinical trials by using MSCs have also been reported in several review articles and meta-analysis [105–107]. Moreover, failure of *in vivo *engraftment of bone marrow (BM)-MSCs into nonhematopoietic tissue has been reported previously [108–110]. Various strategies can be employed to overcome this problem. For instance, in a recent publication, Jin et al. (2011) reported that the 1st passage of mouse BM-MSCs had shown better engraftment and differentiation potential to cardiomyocytes *in vivo*, compared to the 5th passage mouse BM-MSCs [111]. In addition, murine MSCs preconditioned in hypoxic environment showed enhanced skeletal muscle regeneration at day 7 and improved blood flow and vascular formation compared to MSCs maintained in normoxic condition [112]. Furthermore, expression of chemokine receptors CXCR4, CXCR7, and CX3CR1 was upregulated when MSCs were exposed to hypoxia or a reagent that mimics the response to hypoxia [94, 113–115]. These chemokine receptors play an important role in damaged-tissue-specific trafficking and homing of MSCs [113, 115–118]. 

## 4. Biochemical and Molecular Changes due to Hypoxia

O_2_ concentration in the stem cell niche (usually 2–9% O_2_) is considered a driver of cell function [40]. Hypoxia plays a vital role in maintaining homeostasis within the body from the very beginning of embryonic development. It helps facilitate proper embryonic development, maintain stem cell pluripotency, induce differentiation, and regulate the signalling of multiple cascades, including angiogenesis [119]. In hypoxic conditions, usually these functions are regulated by several transcription factors such as hypoxia-inducible factors (HIFs), prolyl-hydroxylases (PHDs), factor-inhibiting HIF-1 (FIH-1), activator protein 1 (AP-1), nuclear factor (NF)-*κ*B, p53, and c-Myc [120]. Although interaction among all of the transcription factors is required for cellular response, HIFs (especially HIF-1) are the key regulators of cellular response to hypoxia [121].

### 4.1. Regulation of Transcription by HIF-1 during Direct Sensing of Changes in Oxygen

**Figure 2:**
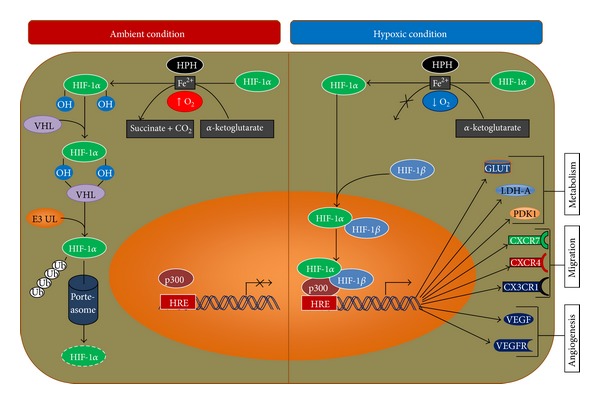
Regulation of transcription by HIF-1 during ambient and hypoxic condition. HIF: hypoxia-inducible factor; HPH: HIF-1 prolyl-hydroxylases; VHL: Von Hippel-Lindau; E3UL: E3 ubiquitin ligase; HRE; hypoxia-response element; GLUT; glucose transporter: LDH: lactate dehydrogenase; PDK, pyruvate dehydrogenase kinase (see text for details).

The HIF-1*β* subunit of a heterodimeric transcription factor HIF-1 (HIF-1*α* and HIF-1*β*) [122, 123] is nonresponsive to oxygen, whereas HIF-1*α* is an oxygen labile protein. Therefore, under ambient condition the HIF-1*α* subunit is usually synthesized and degraded rapidly, whereas under hypoxic conditions, its breakdown is delayed [122, 124]. Degradation of HIF-1*α* under ambient culture condition (Figure 2) is regulated by HIF-1 prolyl-hydroxylases (HPHs) [125]. HIF-1 prolyl-hydroxylases (HPHs) in the presence of O_2_, iron, and *α*-ketoglutarate hydroxylate the proline residues 402 and 564 of the oxygen-dependent degradation domain (ODD) of HIF1*α* [126, 127], which in turn induce a conformational change of HIF*α*, thus allowing Von Hippel-Lindau protein (VHL) to bind with it [62]. Consequently, VHL binds to a complex that serves as E3 ubiquitin ligase (E3UL) and ubiquitinylate HIF-1*α* for degradation in proteasome [63, 128, 129].

In contrast, under hypoxic conditions, the prolyl-hydroxylation process is suppressed due to lack of O_2_ that allows HIF-1*α* accumulation and nuclear translocation to occur [124]. After nuclear translocation, it binds with HIF-1*β* to form the heterodimer. Then the HIF-1 heterodimer binds to a hypoxia-response element (HRE) in the target genes, associated with coactivators such as CBP/p300, and regulates the transcription (Figure 2) of as many as 70 genes involved in metabolism, angiogenesis, invasion/metastasis, and cell fate [130]. 

### 4.2. Reduction of Reactive Oxygen Species by Suppressing Mitochondrial Respiration during Hypoxia

**Figure 3:**
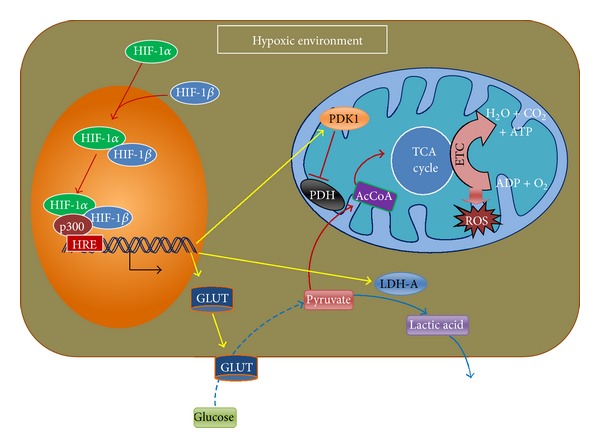
Suppression of mitochondrial respiration by HIF-1*α* in hypoxic environment. HIF: hypoxia-inducible factor; HRE: hypoxia-response element: GLUT: glucose transporter; LDH: Lactate dehydrogenase; PDH: pyruvate dehydrogenase; PDK: pyruvate dehydrogenase kinase; TCA: tricarboxylic acid; ETC: electron transport chain; ROS: reactive oxygen species (see text for details).

Relatively recent discoveries also support the role of HIF-1*α* on metabolic regulation by suppressing mitochondrial respiration. In hypoxic conditions, stabilized HIF-1*α* translocates into the nucleus and binds to HIF-1*β* to form the heterodimer, which in turn binds to the target gene-specific HREs to transcriptionally activate genes that code for glucose transporters (GLUT), glycolytic enzymes, and lactate dehydrogenase-A (LDH-A) to facilitate anaerobic respiration [130, 131]. Besides suppression of mitochondrial respiration, HIF-1*α* promotes the expression of pyruvate dehydrogenase kinase (PDK) that prevents the conversion of pyruvate into acetyl CoA [131] inhibiting the enzymatic activity of pyruvate dehydrogenase (PDH) (Figure 3). This results in the reduction of mitochondrial O_2_ consumption, and as a consequence, the production of reactive oxygen species (ROS) is lowered [132, 133]. In addition, HIF-1*α* in a hypoxic condition causes the production of cytochrome c that also ensures optimum ATP production and cell integrity, by minimizing ROS [134]. 

### 4.3. Induction of Notch Target Genes by Hypoxia

**Figure 4:**
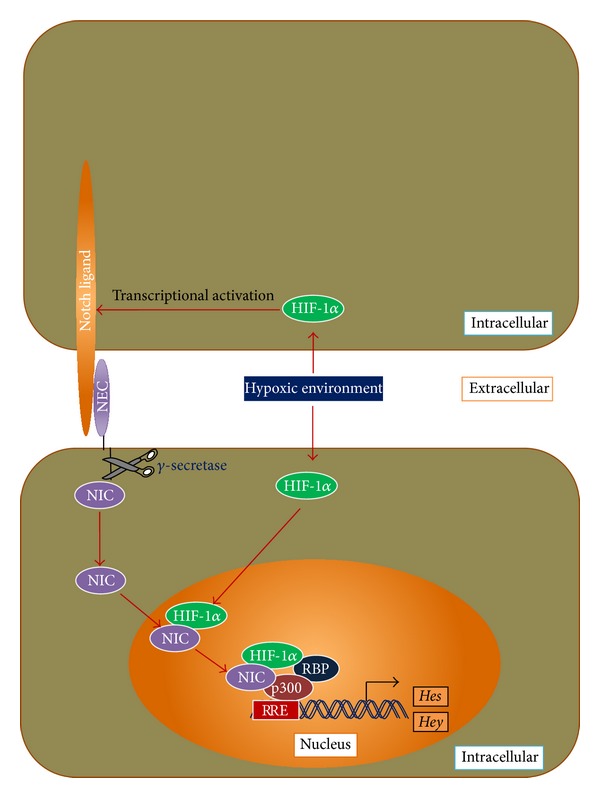
Crosstalk between hypoxia and notch signaling, and regulation of stem cell proliferative gene expression. HIF: hypoxia-inducible factor; NEC: notch extracellular domain; NIC: notch intracellular domain; RBP: recombination-signal binding protein. (Modified from Gustafsson et al., (2005) [34] and Sainson and Harris (2006) [136]; see text for details).

The Notch signaling pathway is an important pathway that regulates the stem cells fate [135]. Crosstalk between hypoxia and activated Notch signaling (Figure 4) has been reported by several researchers [34, 136]. In hypoxic conditions, HIF-1*α* can regulate cell fate by activation of Notch down-stream genes (e.g., *Hes* and *Hey*) necessary to maintain proliferation of stem cells. During this crosstalk, in response to ligand presentation from neighboring cells, Notch receptors undergo proteolytic activation that is mediated by two proteases (tumour necrosis factor and *γ*-secretase). Due to the proteolytic activity, Notch intracellular domain (NIC) is released and translocated into the nucleus. There, NIC binds to HIF-1*α* to build heterodimer which binds to recombination-signal binding protein-Jk (RBP-Jk), CBP/p300 proteins, and RBP-Jk response element (RRE) in the Notch target genes to activate them (e.g., *Hes* and *Hey* genes) [34, 136]. 

### 4.4. Upregulation of Chemokine Receptors by Hypoxia

**Figure 5:**
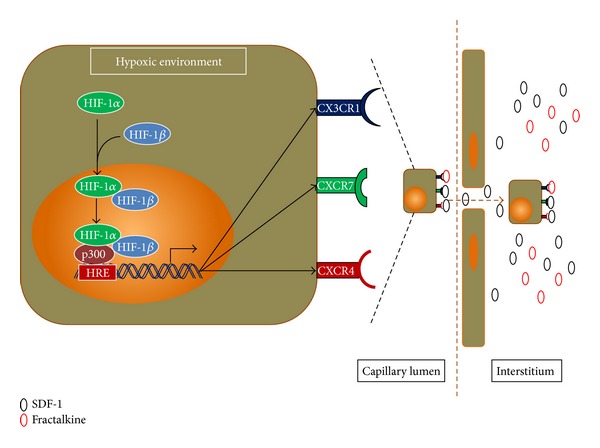
Upregulation of the expression of chemokine receptors by HIF-1*α* in hypoxic environment to facilitate target organ-specific chemotaxis. HIF: hypoxia-inducible factor; HRE: hypoxia-response element (see text for details).

The success of cell-based therapies highly depends upon the engraftment of the transplanted cells. The engraftment of the transplanted cells to the target organ is mediated through interaction between chemotactic factors (released by the organ) and their receptors on the surface of the transplanted cells. Though there are controversies over the expression of chemokine receptors and their migration towards target organs [137], in recent years, several articles have also reported that interaction between chemokines (SDF-1, fractalkine), and their receptors (e.g., CXCR4, CXCR7, and CX3CR1) play a vital role in chemotaxis, viability, and homing of MSCs both *in vitro* and *in vivo* [113, 138]. Moreover, expression of chemokine receptors on MSCs increases in the presence of HIF-1*α* [113]. The above information indicates that HIF1-*α* obtained stability in hypoxic condition prior to it being translocated into the nucleus, where it binds to HIF-1*β* to form the heterodimer. After that, the heterodimer binds to the gene-specific HRE associated with coactivators such as CBP/p300 [130] and upregulates the expression of chemokine receptors CXCR4, CXCR7, and CX3CR1. These chemokine receptors then respond to chemokines (e.g., SDF-1, fractalkine) secreted from diseased tissues or organs that finally facilitate the chemotaxis of the transplanted MSCs to the target site (Figure 5). 

## 5. Hypoxic Culture Conditions as a Solution for MSC-Based Regenerative Therapy 

The above discussions supported the positive role of hypoxic culture environments for MSCs and provided answers to solve problems related to cell-based therapies. In a hypoxic environment, HIF-1*α* prevents the TCA cycle and results in lower ROS (Figure 3). Lower ROS generation resulted in slowing the rate of telomere shortening [139, 140], and as a consequence replicative senescence might be delayed. Moreover, a hypoxic environment upregulates the expression of Notch target genes (e.g., *Hes* and *Hey* genes), responsible for cell proliferation (Figure 4). Therefore, the higher proliferation rate along with more population doubling in hypoxic conditions [37, 38, 92] may be due to the lowered ROS generation and overexpression of Notch target genes (e.g., *Hes* and *Hey*). 

Maintaining genetic stability is another challenge during *in vitro* expansion of MSCs. Increased rates of aneuploidy, double-stranded DNA breakdown, and faster telomere shortening have been reported for MSCs cultured in ambient condition [37]. Gordon et al. (2012) reviewed the causes and consequences behind aneuploidy. They have defined defective spindle assembly checkpoint, centrosome amplification, and merotelic attachments as major causes behind aneuploidy [141]. Moreover, Wang et al. (2012) have described ROS as the causative factor of defective spindle assembly checkpoint, centrosome amplification and merotelic attachments [142]. ROS also acts in acceleration of telomere shortening and DNA breakdown [143, 144]. In addition, correlation between telomere shortening and aneuploidy in embryonic and hepatocellular carcinoma cells has been reported in recently published articles [145, 146]. The above discussion supports that higher ROS production due to the increased mitochondrial respiration during expansion of MSCs in ambient O_2_ concentration (Figure 3) might be the cause behind genetic instability in them. However, during hypoxia, cells go through anaerobic respiration, and as a result lower the ROS concentration within the cells (Figure 3). This might help in reducing the DNA damage, telomere shortening, and aneuploidy which in return may increase the biosafety of stem cell-based therapy. 

Hypoxic culture conditions may also provide a solution for more efficient engraftment. Recently, it has been reported that early passaged mouse BM-MSCs showed better engraftment than late passaged mouse BM-MSCs in *in vivo* model [111]. Moreover, hypoxic preconditioned murine MSCs also showed enhanced skeletal muscle regeneration and improved blood flow and vascular formation compared to MSCs maintained in normoxic condition [112]. Furthermore, hypoxic conditions cause MSCs to grow faster [37] while maintaining a higher proportion of rapidly self-renewing cells [94]. In addition to that, a hypoxic environment increases the expression of chemokine receptors CXCR4, CXCR7, and CX3CR1 [113, 114], and they may facilitate tissue-specific trafficking of MSCs (Figure 5). From the above information, it can be anticipated that adequate numbers of MSCs with a higher fraction of rapidly self-renewing cells and highly expressed chemokine receptors on their surface can be obtained from the early passages of hypoxic cultures, and that MSCs might increase the efficiency of damaged-tissue-specific migration and engraftment following transplantation. Therefore, culturing MSCs in hypoxic conditions can also be considered as a solution for tissue-specific engraftment.

## 6. Conclusion

MSCs have tremendous potential in regenerative medicine. However, poor growth kinetics, genetic instability, and poor engraftment after transplantation are seen as drawbacks in their translation from bench side to bed side. The above information suggests hypoxic culture conditions (2–5% O_2_ concentration) as a promising solution to overcome these problems. Tissue development and regeneration process solely depend upon the sequential steps of stem cell renewal, specialization, and assembly that are coordinated by the cascades of environmental factors in its niche, rather than with one single dominating factor. Thus, success in cell-based regenerative therapies requires a holistic view of stem cell regulation. Besides maintaining MSCs in physiological oxygen condition, there is a need to develop new techniques to analyze *in vivo* conditions of the stem cell niche, so that the appropriate *in vitro* modelling can yield novel information for niche-directed cell-based therapies.

## Figures and Tables

**Table 1:** Oxygen concentration in different organs and tissues.

Name of the tissue or organ	Oxygen concentration	References
Lung parenchyma	4% to 14%	[64, 65]
Circulation	4% to 14%	[63, 66]
Liver	4% to 14%	[64, 67]
Kidneys	4% to 14%	[64, 68]
Heart	4% to 14%	[69, 70]
Brain	0.5% to 8%	[71–73]
Eye (retina, corpus vitreous)	1% to 5%	[74, 75]
Bone marrow	1% to 6%	[35, 36, 76]
Adipose tissue	2% to 8 %	[62]
